# Long‐term health‐related quality of life among men with prostate cancer in the Finnish randomized study of screening for prostate cancer

**DOI:** 10.1002/cam4.3181

**Published:** 2020-06-04

**Authors:** Kirsi Talala, Sirpa Heinävaara, Kimmo Taari, Teuvo L. J. Tammela, Paula Kujala, Ulf‐Håkan Stenman, Nea Malila, Anssi Auvinen

**Affiliations:** ^1^ Finnish Cancer Registry Helsinki Finland; ^2^ Department of Urology University of Helsinki and Helsinki University Hospital Helsinki Finland; ^3^ Faculty of Medicine and Health Technology Tampere University Tampere Finland; ^4^ Department of Surgery Tampere University Hospital Tampere Finland; ^5^ Department of Pathology Fimlab Laboratories Tampere Finland; ^6^ Department of Clinical Chemistry and Hematology Faculty of Medicine University of Helsinki Helsinki Finland; ^7^ Faculty of Social Sciences/Health Sciences Tampere University Tampere Finland

**Keywords:** prostate cancer, quality of life, screening

## Abstract

**Background:**

The long‐term health‐related quality of life (HRQOL) impacts of PCa screening have not been adequately evaluated. We aimed to compare the generic and disease‐specific health‐related quality of life (HRQOL) among men with prostate cancer in the screening arm with the control arm of the PSA‐based prostate cancer screening trial in up to 15 years of follow‐up.

**Materials and methods:**

This study was conducted within population‐based Finnish Randomized Study of Screening for Prostate Cancer (FinRSPC). During 1996‐1999 80,458 men were randomized to the serum prostate‐specific antigen (PSA) screening arm (SA, N = 32 000) and the control arm (CA, N = 48 458). Men in the screening arm were screened at 4‐year intervals until 2007. HRQOL questionnaires were delivered to newly diagnosed prostate cancer patients in the screening and control arm 1996‐2006 (N = 5128) at the time of diagnosis (baseline), at 3‐month, 12‐month and 5, 10, and 15‐year follow‐up. Validated UCLA Prostate Cancer Index (UCLA‐PCI) and RAND 36‐Item Health Survey were used for HRQOL assessment. The data were analyzed with a random effects model for repeated measures.

**Results:**

At baseline, men with prostate cancer in the screening arm reported better Sexual Function, as well as less Sexual and Urinary Bother. Long‐term follow‐up revealed slightly higher HRQOL scores in the screening arm in prostate cancer specific measures at 10‐year post diagnosis, but the differences were statistically significant only in Urinary Bother (UCLA‐PCI score 77.9; 95% CI 75.2 to 80.5 vs. 70.9; 95% CI 66.8 to 74.9 *P = .005*). The generic HRQOL scores were comparable between the trial arms. The overall differences in disease‐specific or generic HRQOL scores by trial arm did not vary during the follow‐up.

**Conclusion:**

No major differences were observed in HRQOL in men with prostate cancer between the prostate cancer screening and control arms during five to 15‐year follow‐up.

## Graphical abstract

Our study contributes to the evaluation of the benefits and harms of prostate cancer screening on long‐term quality of life. The results suggest that screening does not materially affect long‐term quality of life in men with prostate cancer. Model‐based mean scores (95% CIs) in Urinary Bother by arm of the FinRSPC.

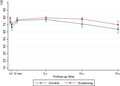

## 1 INTRODUCTION

Prostate cancer (PCa) is the most common cancer and one of the leading causes of cancer death in men in Western countries.^1^ Long‐term survival following PCa diagnosis is good; age‐standardized 5‐year survival is in the range 70%‐100% in most countries.^2^ PCa is the main global contributor to years lived with cancer disability^3^ and men with clinically detected PCa have shown to experience treatment‐related adverse effects at 5 years,^4^ and even up to 10‐15 years after diagnosis.^5^, ^6^

European Randomized Study of Screening for Prostate Cancer (ERSPC) has shown reduced incidence of advanced disease,^7^ and a 20% relative reduction in PCa mortality over 16‐year follow‐up.^8^ Hence screening for PCa could potentially offer major benefits, and improve quality of life in men with screen‐detected PCa. However, frequent overdiagnosis and treatment of cancers that would not go on to cause symptoms or death may offset any such benefits.^9^ Evaluation of the long‐term health‐related quality of life (HRQOL) effects of screening is important for the overall evaluation regarding PCa screening.^10^, ^11^

Few studies have reported long‐term quality of life outcomes in PCa screening trials and even those studies have been limited by their cross‐sectional design and absence of pretreatment baseline functioning HRQOL assessment. A previous analysis in the Finnish Randomized Study of Screening for Prostate Cancer (FinRSPC) using data from the year 2011 with median 6.7 years (control arm) and 8 years (screening arm) of follow‐up suggested slightly higher generic HRQOL scores in the screening arm for men diagnosed with PCa (as measured by 15D, EQ‐5D and SF‐6D; statistically significant only for EQ‐5D), than for such men in the control arm. No differences were found between the arms for men in the trial subsample free of PCa.^12^ The current study differs from the earlier publication in that the data for this study was collected at regular intervals after diagnosis which permits more robust interpretation of the results, whereas the previously published study was not conducted at set points after diagnosis, but at fixed points after the start of the trial. In addition, our current study has data for both generic (RAND‐36) and disease‐specific (UCLA‐PCI) HRQOL. In the US Prostate, Lung, Colorectal, and Ovarian (PLCO) Cancer Screening Trial no clear difference was found between trial arms in disease‐specific quality of life scores among PCa survivors, suggesting that screening detection does not affect urinary, sexual, and bowel functioning at 5 to 10 years after diagnosis.^13^

Majority of patients diagnosed in the PCa screening will live at least 10‐15 years after diagnosis, however, research evidence on long‐term HRQOL effects of PCa‐screening is still scarce. This study aims to broaden the knowledge of the overall impact of prostate‐specific antigen (PSA)‐based PCa screening at population level by comparing both generic (RAND‐36) and disease‐specific (UCLA‐PCI) HRQOL among PCa patients in the screening arm and control arm in a randomized trial with 15‐year follow‐up.

## 2 MATERIAL AND METHODS

The study is based on the population‐based Finnish Randomized Study of Screening for Prostate Cancer (FinRSPC), which is the largest component of the multicenter ERSPC trial. The Finnish trial recruited 80 458 men during 1996‐1999 at two screening centers, Helsinki and Tampere. Annually, 8000 men aged 55, 59, 63, or 67 years were randomized to the screening arm (N = 32 000), and the remaining men (N = 48 458) formed the control arm. Men in the screening arm were screened at 4‐year intervals until 2007. Trial protocol have been described previously in detail.^14^ In the present study, men with PCa in the screening arm and control arm were compared on an intention‐to‐treat basis, ie regardless of screening compliance, to focus on population level effect of screening. 

**Figure 1:**
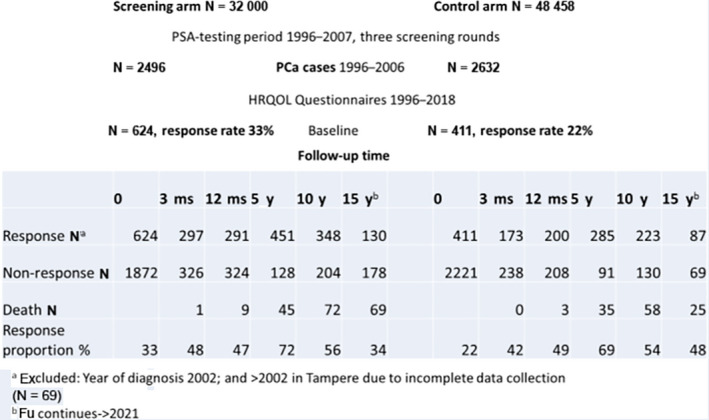
Study population and data collection for the HRQOL data in the Finnish Randomized Study of Screening for Prostate Cancer (FinRSPC)

A total of 5218 incident PCa cases in both arms were identified from the Finnish Cancer Registry during 1996‐2006. HRQOL questionnaires were delivered at the time of diagnosis (baseline), prior to primary treatment, at 3 months, 12 months, 5, 10, and 15 years after the PCa diagnosis (Figure 1).

HRQOL was evaluated using both generic and disease‐specific domains. UCLA Prostate Cancer Index (UCLA‐PCI) has demonstrated validity as a disease‐specific HRQOL measure in men with PCa.^15^ It consists of six scales; Urinary Function, Sexual Function, Bowel Function, and a Bother assessment for Urinary, Bowel and Sexual domains.

The RAND 36‐Item Health Survey (also known SF‐36) is a validated and widely used generic health instrument.^16^ It consists of eight scales: Physical Functioning, Role‐Physical, Role‐Emotional, Bodily Pain, General Health, Vitality, Social Functioning, and Mental Health.

The RAND‐36 and UCLA‐PCI scales both range from 0 to 100 after re‐scoring, with a score of 100 representing optimal health, normal functioning, or no bother. Differences in the UCLA‐PCI^17^ and RAND scales^18^ of 10 points are suggested by their authors to be clinically meaningful for 0 to 100 scales.

Background variables included age at diagnosis, screening center (Helsinki vs Tampere), comorbidity,^19^ PCa tumor risk group, primary treatment group, and national registry data for educational level, socioeconomic, and marital status. The European Association of Urology PCa tumor risk group classification^20^ was used based on PSA, Gleason scores and TNM stage.

### 2.1 Statistical analyses

Background variables were compared between the trial arms using χ^2^ tests. In the main analyses, we analyzed changes over time in HRQOL scores in the two arms using a linear random effects model with population‐averaged estimator with exchangeable correlation structure, which can take into account the statistical dependence among observations in repeated measures. Missing values were not imputed, as less than 5% of the cases were missing and considered minor. We applied the inverse probability weighting method to reduce biases due to imperfections in the sample related to noncoverage and unit nonresponse. It involves weighting each participant's contribution to the estimation according to how likely they were, compared to the target population (ie diagnosed PCa cases), to be complete records based on poststratification for arm, age, screening center, comorbidity, educational level, prostate cancer risk group, and the interaction term for the year of diagnosis and arm. All analyses were adjusted for the assigned sampling weights.

**Figure 2:**
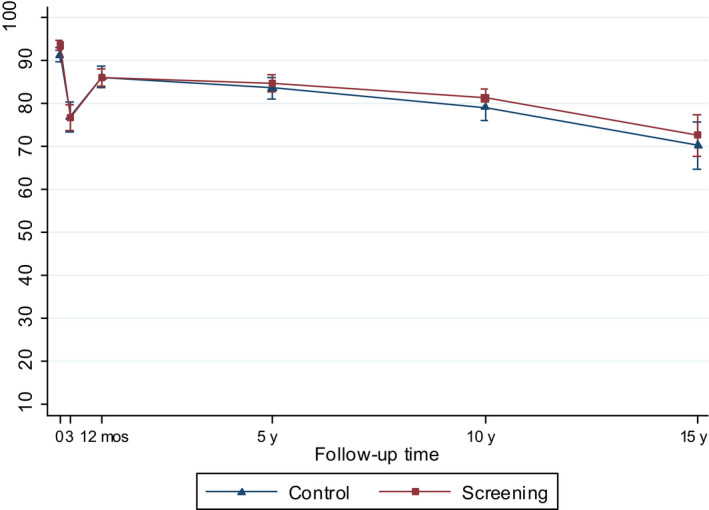
Model‐based mean scores (95% CIs) in Urinary Function by arm of the FinRSPC

**Figure 3:**
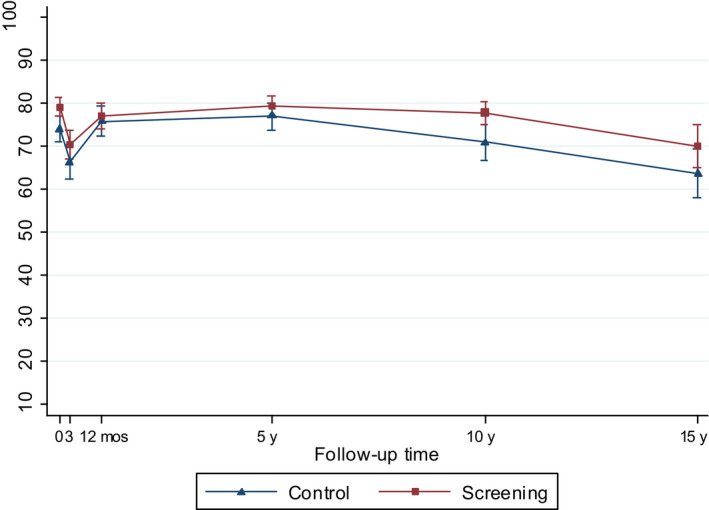
Model‐based mean scores (95% CIs) in Urinary Bother by arm of the FinRSPC

**Figure 4:**
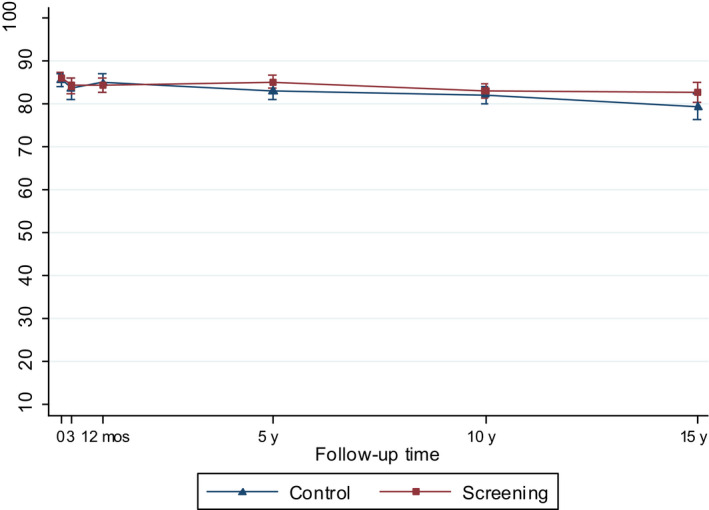
Model‐based mean scores (95% CIs) in Bowel Function by arm of the FinRSPC

**Figure 5:**
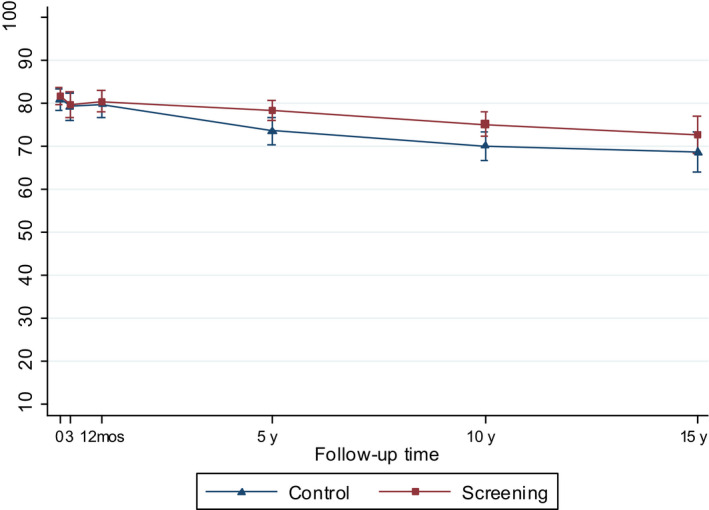
Model‐based mean scores (95% CIs) in Bowel Bother by arm of the FinRSPC

**Figure 6:**
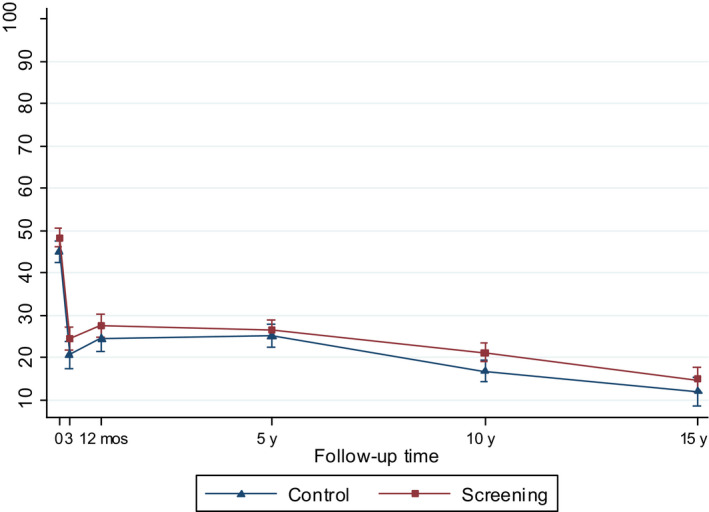
Model‐based mean scores (95% CIs) in Sexual Function by arm of the FinRSPC

**Figure 7:**
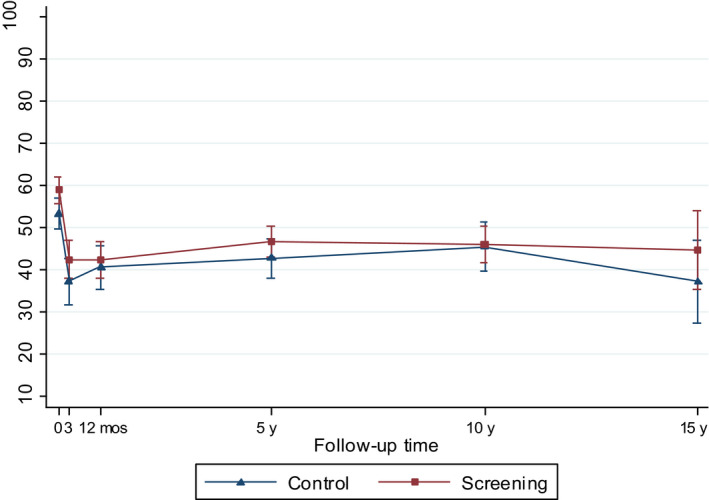
Model‐based mean scores (95% CIs) in Sexual Bother by arm of the FinRSPC

All models included screening center, sampling weights, main effects of trial arm, and follow‐up time, and their interaction term in order to assess whether the effect of screening varied over time, and interaction term for age and time. Variables were treated as time‐invariant based on the measure at the time of diagnosis. To avoid overadjustment, ie adjustment for causal intermediates of the intervention examined, the models did not include tumor risk group or treatment, which are likely affected by screening, as this would be expected to bias the results toward the null. To facilitate interpretation, we presented the main results using predictive margins and their 95% confidence intervals (CI) (Figures 2, 3, 4, 5, 6, 7) and tested statistical difference with marginal effects *P* values. Predictive margins are statistical measures computed from predictions given by a regression model; individual predictive values are used to calculate the mean predictive values, ie predictive margins, while adjusting for the values of the covariates.^21^

We conducted sensitivity analyses with generalized estimating equations models with robust standard errors, unstructured correlations, and maximum likelihood estimations as alternative approaches for analyzing the data. Sensitivity analyses resulted to equivalent results to the models we used. We also conducted per protocol sensitivity analyses comparing screen‐detected cancers to other detection methods. In these analyses results were not converted, although in some disease‐specific HRQOL variables differences were more pronounced, compared to the original analyses. All *P* values were two‐sided and a significance level of 0.05 was applied in statistical tests. We carried out all the statistical analyses using Stata 14.0.^22^

## 3 RESULTS

During 1996‐2006, 5128 new PCa cases were diagnosed in the study population (Figure 1). Data collection failed for the year 2002 for both centers (response proportion 2,5%), and after 2002 in the Tampere center. These data were excluded, and 624 eligible cases remained in the screening arm (response proportion 33%) and 411 in the control arm (response proportion 22%) with an overall response proportion of 27%. The mean follow‐up time was 8.3 years, with a median of 10 years.

Compared to the screening arm, respondents in the control arm were significantly older, had lower proportion of low‐risk group cancers (*P < .01*), and were more commonly from Helsinki screening center (*P < .001*) (Table 1). There were no differences between the arms in any other sociodemographic variables or comorbidity. Surgery and active surveillance were more frequent primary treatments in the screening arm, while radiotherapy and hormonal therapy were more common in the control arm (*P < .001*).

**Table 1:** Descriptive characteristics of the men with prostate cancer at baseline (N = 1035) within the Finnish Randomized Study of Screening for Prostate Cancer (FinRSPC)

Variable	Screening arm N % 624 (60)	Control arm N % 411 (40)	*P* value	Total N % 1035 (100)
Age group at the time of diagnosis			<.001	
54–59 years	102 (16)	33 (8)		135 (13)
60–64 years	217 (35)	112 (27)		329 (32)
65–69 years	215 (34)	169 (41)		384 (37)
70+ years	90 (14)	97 (23)		187 (18)
Marital status			.17	
Married	486 (78)	319 (78)		805 (78)
Not married	31 (5)	31 (7)		62 (6)
Not known	107 (17)	61 (15)		168 (16)
Educational level			.73	
Highest	241 (39)	163 (40)		404 (39)
Intermediate	131 (21)	78 (19)		209 (20)
Lowest	252 (40)	170 (41)		422 (41)
Socioeconomic status			.17	
Upper‐level employee	48 (8)	23 (6)		71 (7)
Higher‐level employee	40 (6)	17 (4)		57 (6)
Self‐employed person	21 (3)	15 (4)		36 (3)
Manual worker	39 (6)	25 (6)		64 (6)
Pensioners	438 (70)	316 (77)		754 (73)
Unemployed	29 (5)	13 (3)		42 (4)
Not known	9 (1)	2 (0.5)		11 (1)
Comorbidity			.311	
0 Conditions	528 (85)	338 (82)		866 (84)
≥1 Conditions	96 (15)	73 (18)		169 (16)
Screening center				
Helsinki	450 (72)	328 (80)	.005	778 (75)
Tampere	174 (28)	83 (20)		257 (25)
Tumor risk group				
Lowest	303 (49)	107 (26)	<.001	422 (41)
Intermediate	174 (28)	157 (38)		331 (32)
Highest	143 (23)	138 (34)		281 (27)
Not known	4 (0.5)	9 (2)		1 (‐)
Primary treatment				
Surgery	245 (39)	118 (29)	<.001	363 (35)
Radiotherapy	194 (31)	188 (46)		382 (37)
Hormonal therapy	51 (8)	62 (15)		113 (11)
Active surveilance	133 (21)	43 (10)		176 (17)
No treatment	1(‐)	‐		1 (0.1)

In the nonresponse analyses (data not shown), nonparticipation was more likely in the higher risk group cancer patients and patients with hormonal therapy treatment in both arms, and among upper‐level employees in the control arm. None of other socioeconomic status dimensions, marital status or comorbidity were associated with nonresponse. Radical prostatectomy patients were over‐representative in the HRQOL sample compared to the total prostate cancer cohort in both arms, as well as the age‐group 60‐64 years in the screening arm, and the age‐group 65‐69 years and intermediate‐risk disease group in the control arm.

### 3.1 Prostate cancer‐specific quality of life

The model‐based mean values for each UCLA‐PCI scale present the changes in HRQOL scores over time in the two trial arms (Figures 2, 3, 4, 5, 6, 7). At baseline, the patients in the screening arm showed statistically significantly higher scores, ie less bother, than those in the control arm in Urinary Bother (79.2; 95% CI 77.1 to 81.3 vs 74.0; 95% CI 71.0 to 76.9; *P = .005*) (Figure 3). The Urinary and Sexual Function, and Sexual Bother scores were also nonsignificantly higher in the screening arm (Figures 2, 6 and 7). The HRQOL scores in bowel domains did not differ by trial arm at baseline or in short‐term follow‐up.

Urinary Function, Sexual Function, and Sexual Bother scores declined steeply (more than 10 points) at 3 and 12 months after diagnosis in both arms. Urinary Function showed an improvement at 12 months. In short‐term follow‐up, the differences in all disease‐specific measures between the trial arms tended to decrease from the baseline.

At 5 to 15‐years, the men in the screening arm showed a tendency toward somewhat higher disease‐specific HRQOL scores in all domains compared with those in the control arm. The differences were statistically significant, however, only in Urinary Bother; at 10‐year follow‐up men in the screening arm reported higher scores, ie less bother (77.9; 95% CI 75.2 to 80.5), relative to the control arm (70.9; 95% CI 66.8 to 74.9; *P = .005*)(Figure 3). In Bowel Bother, higher scores emerged in the screening arm only at 5 to 15‐years postdiagnosis (Figure 5). However, the interaction term of trial arm and time was not statistically significant in any domain, indicating lack of systematic changes over time for the difference between the arms. In Bowel Function, there were no marked changes, or differences between arms at any time point (Figure 4).

Unadjusted HRQOL scores together with distribution of one item of each scale are presented by arm in the Table A1.

Patients in the screening arm with low risk tumors reported significantly better Sexual Function at baseline and less Urinary Bother at 10‐year follow‐up compared to control arm (data not shown). However, no statistically significant interaction with trial arm was found overall. Sensitivity analyses with adjustment for both tumor risk group and primary treatment showed no clear reduction in the differences between the arms in HRQOL.

### 3.2 Generic health‐related quality of life

At baseline, the generic HRQOL scores did not differ significantly between the arms, although the mean scores for the screening arm were slightly (1‐3 points) higher on all domains (Table 2). In contrast, at 3 to 12‐month follow‐up, patients in the screening arm reported similar or nonsignificantly lower scores than the control arm. There was decline at the 3‐month postdiagnosis compared to the baseline especially in Role‐Emotional (by 6.9 points), and in Role‐Physical (by 7.4 points) functioning. At 5 to 15‐year follow‐up, the screening arm had similar or higher mean RAND‐36 scores than the control arm, though the differences were not significant in any of the generic quality of life dimensions. Furthermore, there was no significant interaction between trial arm and time on any domains of generic HRQOL.

**Table 2:** Adjusted model‐based mean scores (95% CIs) in generic quality of life (RAND‐36) measures by trial arm in the Finnish Randomized Study of Screening for Prostate Cancer (FinRSPC)

Screening arm							Control arm					
	Baseline N = 624	3mo N = 297	12mo N = 291	5yrs N = 451	10yrs N = 348	15yrs N = 130	Baseline N = 411	3mo N = 173	12mo N = 200	5yrs N = 285	10yrs N = 223	15yrs N = 87
Physical Functioning	82 (80‐84)	80 (78‐82)	80 (78‐82)	76 (74‐79)	70 (67‐73)	61 (56‐65)	81 (79‐83)	82 (80‐85)	82 (79‐85)	76 (73‐79)	68 (65‐71)	59 (54‐63)
Role‐physical	73 (70‐76)	65 (60‐70)	67 (63‐72)	65 (61‐69)	55 (50‐60)	42 (33‐51)	72 (68‐76)	71 (66‐76)	76 (72‐81)	67 (62‐72)	57 (51‐62)	40 (29‐52)
Role‐emotional	74 (70‐77)	67 (62‐71)	72 (68‐77)	71 (67‐75)	66 (61‐70)	58 (51‐66)	70 (66‐74)	72 (67‐77)	78 (73‐83)	73 (68‐78)	65 (60‐70)	57 (49‐65)
Bodily Pain	81 (79‐83)	82 (79‐84)	80 (77‐83)	79 (77‐82)	76 (73‐78)	69 (64‐73)	82 (79‐84)	82 (79‐85)	83 (80‐86)	80 (77‐82)	73 (69‐76)	71 (66‐76)
General Health	58 (56‐59)	58 (56‐61)	57 (55‐59)	56 (54‐57)	53 (51‐55)	49 (46‐53)	57 (55‐59)	59 (57‐62)	59 (57‐62)	57 (54‐59)	50 (48‐53)	48 (44‐53)
Vitality	67 (66‐69)	67 (64‐70)	69 (67‐72)	67 (65‐69)	63 (61‐66)	57 (53‐62)	67 (65‐70)	68 (65‐71)	69 (66‐71)	65 (63‐68)	61 (58‐64)	57 (51‐62)
Social Functioning	83 (81‐85)	80 (77‐83)	84 (81‐86)	84 (82‐86)	80 (78‐83)	74 (70‐78)	82 (80‐85)	81 (78‐83)	84 (81‐87)	83 (80‐86)	79 (76‐82)	74 (69‐78)
Mental Health	76 (74‐77)	76 (74‐78)	78 (76‐80)	77 (75‐78)	76 (74‐78)	72 (68‐76)	74 (72‐76)	77 (74‐79)	77 (74‐79)	78 (76‐80)	75 (73‐78)	74 (69‐79)

## 4 DISCUSSION

The long‐term HRQOL impacts of PCa screening have not been adequately evaluated. Our results revealed only minor differences in disease‐specific HRQOL at 5‐15 years, with generally slightly higher mean scores in the screening arm, though not of the magnitude that have been considered clinically meaningful (over 10 points). No substantial or consistent differences between the trial arms emerged in generic HRQOL.

Opportunistic screening, ie PSA screening outside a screening program, will dilute the effect of screening. We compared groups by the trial arm allocated by randomization (intention‐to‐screen analysis). PSA testing has been shown to be common in the control arm of the FinRSPC trial.^23^At least one PSA test was performed for 18% of the men in the control arm by four years, and it reached 48% by eight years. The mortality reduction in Finnish trial alone has been small,^24^ likely reflecting both contamination and shorter screening period with fewer screening rounds than in the ERSPC Göteborg and ERSPC Rotterdam.^25^ PLCO trial showed no mortality reduction likely due to widespread contamination, and low biopsy compliance.^10^, ^11^ In centers with larger mortality effect, the impact of screening on quality of life may also be greater. HRQOL results in the PLCO trial indicated no substantial long‐term differences between arms in disease‐specific measures.^13^ Contamination has likely diluted differences between the arms, and this can be expected to also affect the current HRQOL results.

Overdiagnosis, detecting cases that would not have been diagnosed in the absence of screening, has been estimated to comprise 21%‐50% of all screen‐detected PCas.^9^, ^10^ Overdiagnosis, as well as lead‐time and length time bias affect the distribution of prognostic factors among screen‐detected cases. In the present study, low‐risk cancers (T1‐T2, Gleason < 7, PSA < 10) were more common in the screening arm compared to the control arm (45% vs 30%) and the distribution was even more pronounced among the quality of life‐survey respondents (49% vs 26%). However, adjustment for the tumor risk group did not substantially affect the results, suggesting that the influence of overdiagnosis on our findings is limited. Based on simulation model prediction, Heijnsdijk and colleagues^26^ concluded that overall benefit of PSA screening from averted deaths and advanced cancers was decreased by loss of quality‐adjusted life‐years (QALYs) caused by overdiagnosed cancers.

Recent reviews in PCa treatment have concluded that adverse sexual, urinary, and bowel effects occur more commonly in men with active treatments compared to conservative management (eg active surveillance, watchful waiting).^4^, ^10^ Fenton and colleagues also concluded, based mainly on studies among clinically detected patients with ≤5 years follow‐up, that despite difficulties in PCa‐specific domains of functioning, active treatments for PCa did not clearly compromise generic quality of life or physical, or mental health status compared with conservative management.^10^ Within the ERSPC trial, after accounting for disease and patient characteristics, trial arm had only a minor role in treatment choice compared to other variables.^27^ We conducted supplementary analyses with adjustment for treatment, which did not materially affect the HRQOL differences between the trial arms.

Our study had some limitations. Firstly, the overall response proportion did not exceed 22% of the eligible men, which suggest a possibility of selection bias and may challenge the generalizability of our findings. After exclusion of data due to failed procedures at data collection response proportion was 27%, and we used weighting to improve the representativeness of the study population. However, the lack of statistical power may have prevented us from observing differential time trends between the screening and control arms. In the Sexual Bother question, ’sexual function’ was translated as ‘sexual life’. Therefore, responses may be related to sexual function, but also other sexual problems.

The major strength of our study was the material collected within a randomized screening trial, which maximizes the comparability of the groups and can minimize confounding and selection bias. To our knowledge, this study is the first PSA screening trial evaluating HRQOL in a longitudinal prospective study design including baseline assessment, with an exceptionally long follow‐up including both PCa‐specific and generic HRQOL measures. Baseline measurement is necessary in longitudinal studies to evaluate changes in HRQOL over time, and to distinguish impairments from those present already at baseline. Our results and conclusions were independent on the chosen statistical methods. Finally, we were able to obtain data on background sociodemographic characteristics in a comprehensive fashion from national registers.

In conclusion, our long‐term evaluation of disease‐specific and generic HRQOL did not reveal any large or systematic differences in men with PCa between the screening and control arms of the Finnish Randomized Study of Screening for Prostate Cancer.

## CONFLICT OF INTERESTS

AA had financial support from Cancer Foundation Finland, AA and TLJT from Academy of Finland (grant #260931) and TLJT from Pirkanmaa Hospital District Competitive Research Funding (Grant No 9V065). Outside the submitted work: AA declares receiving a fee for expert consultation by Epid Research Inc. K.Taari declares receiving research funding from Medivation/Astellas/Pfizer, Orion and Myovant. TLJT declares receiving grants from Sigrid Juselius Foundation, personal fees from Bayer AG, Janssen‐Cilag and Astellas. PK declares receiving travel costs to a meeting in Finland from Company Amgen. All other authors have no conflicts to disclose.

## AUTHOR CONTRIBUTIONS

AA and TLJT are principal investigators, who conceived and designed the original proposal for the study and obtained trial funding. K.Talala, SH and AA contributed to the planning and reporting of the analyses. K.Talala worked the data, conducted the statistical analyses and prepared first draft of the manuscript. All authors contributed to the interpretation of data,commented on the contents, revised on the manuscript and approved the final version for submission.

## ETHICAL STATEMENT

The Ethics committee of the Pirkanmaa Hospital District evaluated the study protocol (tracking number R10167). National Institute for Health and Welfare (Dnro THL/1601/5.05.00/2015) and Statistics Finland (TK‐53‐1330‐18) has approved research permission.

## Data Availability

No additional data available.

## Appendix 1

**Table A1:** Unadjusted (weighted) mean values (SD) for prostate cancer‐specific functioning (UCLA PCI) and generic HRQOL (RAND‐36) (score 100 indicating optimal health), and distribution (%) of one item of each scale

** Screening arm**							**Control arm**					
	Baseline N = 624	3mos N = 297	12mos N = 291	5yrs N = 451	10yrs N = 348	15yrs N = 130	Baseline N = 411	3mos N = 173	12mos N = 200	5yrs N = 285	10yrs N = 223	15yrs N = 87
**Urinary Function**	**93 (15)**	**74 (33)**	**86 (25)**	**84 (24)**	**81 (25)**	**74 (28)**	**91 (14)**	**79 (21)**	**87 (18)**	**85 (19)**	**80 (21)**	**71 (24)**
***Urinary control***	N (%)	N (%)	N (%)	N (%)	N (%)	N (%)	N (%)	N (%)	N (%)	N (%)	N (%)	N(%)
*No control*	3 (0.4)	4 (1)	0 (0)	2 (0.4)	1 (0.3)	3 (3)	5 (1)	5 (3)	1 (0.4)	1 (0.4)	6 (3)	5 (5)
*Frequent dribbling*	14(2)	41(14)	19(7)	28(6)	28(8)	18(14)	19 (4)	16 (8)	9 (4)	20 (7)	20 (10)	13 (15)
*Occasional dribbling*	136 (22)	121 (41)	94 (32)	170 (37)	159 (46)	57 (44)	97 (25)	70 (40)	73 (36)	110 (39)	101 (47)	36 (43)
*Total control*	469 (75)	130 (43)	176 (60)	247 (55)	155 (44)	51 (38)	287 (69)	79 (47)	116 (58)	153 (54)	92 (40)	31 (36)
**Urinary Bother**	**79 (29)**	**69 (35)**	**77 (30)**	**80 (30)**	**78 (29)**	**73 (31)**	**74 (24)**	**67 (23)**	**76 (21)**	**78 (22)**	**72 (26)**	**64 (25)**
	N (%)	N (%)	N (%)	N (%)	N (%)	N (%)	N (%)	N (%)	N (%)	N (%)	N (%)	N (%)
*Big problem*	10 (1)	17 (6)	6 (2)	14 (3)	8 (2)	5 (5)	12(3)	6(4)	5(2)	7(2)	14(7)	6 (6)
*Moderate problem*	41 (7)	34 (11)	29 (11)	30 (6)	24 (6)	14 (11)	38 (10)	21 (11)	12 (7)	18 (7)	15(7)	16 (19)
*Small or very small*	263 (42)	147 (49)	118 (42)	172 (41)	155 (45)	54 (43)	194 (47)	99 (36)	101 (52)	41 (45)	108 (50)	41 (49)
*No problem*	305 (49)	99 (34)	137 (46)	232 (50)	151 (43)	55 (41)	166 (40)	44 (26)	79 (38)	132 (46)	84 (36)	23 (26)
**Bowel Function**	**86 (17)**	**84 (19)**	**85 (16)**	**85 (18)**	**84 (17)**	**84 (15)**	**86 (13)**	**84 (14)**	**85 (14)**	**83 (13)**	**83 (13)**	**78 (14)**
***Loose stool***	N (%)	N (%)	N (%)	N (%)	N (%)	N (%)	N (%)	N (%)	N (%)	N (%)	N (%)	N(%)
*Always*	6 (1)	4 (2)	4 (1)	4 (1)	1 (0.3)	2 (1)	7 (2)	4 (2)	2 (1)	2 (1)	5 (2)	0 (0)
*Usually*	56 (10)	26 (9)	28 (11)	39 (10)	23 (7)	9 (9)	39 (10)	13 (8)	15 (8)	27 (11)	8 (4)	6 (7)
*Half the time*	52 (8)	24 (8)	27 (9)	31 (7)	29 (8)	12 (9)	26 (6)	11 (8)	23 (11)	18 (7)	27 (12)	8 (10)
*Rarely*	349 (55)	155 (51)	160 (56)	253 (56)	205 (59)	75 (58)	212 (51)	93 (51)	109 (53)	172 (58)	130 (60)	53 (64)
*Never*	158 (25)	87 (30)	72 (23)	120 (26)	81 (23)	31 (22)	124 (30)	51 (30)	47 (26)	64 (22)	51 (22)	18 (19)
**Bowel Bother**	**82 (27)**	**80 (28)**	**82 (26)**	**79 (29)**	**76 (29)**	**74 (27)**	**81 (20)**	**80 (20)**	**80 (20)**	**74 (22)**	**72 (21)**	**70 (19)**
	N (%)	N (%)	N (%)	N (%)	N (%)	N (%)	N (%)	N (%)	N (%)	N (%)	N (%)	N(%)
*Big problem*	10 (2)	3 (2)	2 (0.6)	7 (1)	2 (1)	2 (2)	3 (1)	0 (0)	1 (0.4)	6 (2)	2 (1)	0 (0)
*Moderate problem*	22 (4)	12 (5)	13 (5)	37 (8)	27 (8)	9 (8)	29 (7)	14 (8)	14 (7)	25 (9)	20 (10)	9 (12)
*Small problem*	80 (13)	40 (13)	38 (14)	46 (11)	55 (16)	20 (16)	36 (9)	19 (11)	21 (12)	42 (15)	39 (18)	17 (21)
*Very small problem*	171 (27)	95 (31)	85 (29)	143 (32)	115 (32)	51 (38)	137 (33)	56 (32)	68 (34)	96 (36)	93 (44)	38 (44)
*No problem*	340 (54)	145 (48)	153 (52)	217 (48)	141 (41)	47 (35)	203 (49)	83 (47)	94 (46)	116 (37)	67 (28)	22 (23)
**Sexual Function**	**49 (30)**	**23 (28)**	**29 (30)**	**28 (29)**	**24 (26)**	**18 (20)**	**44 (23)**	**20 (19)**	**25 (20)**	**26 (20)**	**19 (18)**	**12 (13)**
***Ability to function*** ***sexually***	N (%)	N (%)	N (%)	N (%)	N (%)	N (%)	N (%)	N (%)	N (%)	N (%)	N (%)	N(%)
*Very poor*	120 (20)	165 (56)	131 (46)	200 (46)	156 (46)	77 (59)	96 (24)	100 (57)	100 (52)	134 (48)	124 (58)	61 (72)
*Poor*	138 (22)	66 (22)	76 (25)	120 (26)	98 (27)	37 (28)	114 (28)	48 (30)	43 (21)	82 (29)	58 (26)	16 (19)
*Fair*	226 (36)	46 (16)	55 (18)	99 (21)	65 (17)	10 (8)	133 (31)	18 (10)	43 (21)	49 (16)	26 (11)	4 (4)
*Good*	105 (17)	15 (5)	23 (8)	20 (4)	17 (5)	3 (2)	55 (13)	5 (2)	8 (4)	14 (5)	9 (4)	2 (3)
*Very good*	21 (3)	2 (0.6)	2 (0.6)	6 (1)	0 (0)	0 (0)	7 (2)	0 (0)	1 (0.4)	2 (1)	0 (0)	0 (0)
**Sexual Bother**	**59 (40)**	**42 (45)**	**42 (43)**	**46 (44)**	**46 (44)**	**46 (45)**	**54 (31)**	**40 (32)**	**41 (34)**	**45 (34)**	**48 (36)**	**39 (35)**
	N (%)	N (%)	N (%)	N (%)	N (%)	N (%)	N (%)	N (%)	N (%)	N (%)	N (%)	N(%)
*Big problem*	84 (15)	95 (36)	83 (31)	122 (29)	97 (29)	43 (35)	74 (18)	67 (37)	69 (37)	89 (32)	61 (30)	40 (47)
*Moderate problem*	82(13)	40 (13)	58 19)	65 (15)	54 (16)	17 (12)	70 (17)	24 (13)	22 (11)	44 (17)	39 (18)	7 (8)
*Small problem*	126 (21)	47 (16)	44 (14)	72 (15)	49 (13)	10 (7)	82 (19)	28 (18)	32 (16)	38 (12)	19 (8)	3 (4)
*Very small problem*	132 (20)	47 (14)	47 (15)	89 (18)	58 (17)	19 (14)	76 (19)	23 (12)	30 (14)	46 (15)	29 (13)	13 (16)
*No problem*	179 (29)	59 (19)	50 (18)	93 (20)	75 (22)	35 (27)	100 (24)	27 (17)	38 (19)	64 (22)	65 (29)	20 (22)
**Physical Functioning**	**82 (23)**	**81 (23)**	**80 (24)**	**79 (26)**	**75 (29)**	**69 (28)**	**81 (19)**	**81 (17)**	**81 (21)**	**78 (19)**	**72 (22)**	**63 (23)**
**Role‐physical**	**74 (42)**	**67 (46)**	**67 (46)**	**69 (46)**	**63 (47)**	**55 (43)**	**70 (33)**	**67 (31)**	**72 (34)**	**69 (34)**	**62 (36)**	**50 (35)**
**Role‐emotional**	**73 (43)**	**69 (44)**	**72 (43)**	**73 (44)**	**71 (44)**	**66 (41)**	**70 (34)**	**70 (31)**	**76 (32)**	**74 (33)**	**69 (33)**	**63 (33)**
**Bodily Pain**	**81 (25)**	**81 (25)**	**80 (25)**	**79 (28)**	**78 (25)**	**72 (26)**	**82 (19)**	**82 (17)**	**82 (18)**	**80 (22)**	**75 (22)**	**72 (19)**
**General Health**	**58 (23)**	**60 (23)**	**58 (23)**	**58 (24)**	**57 (23)**	**55 (21)**	**57 (17)**	**58 (17)**	**59 (16)**	**58 (17)**	**53 (17)**	**51 (15)**
**Vitality**	**67 (26)**	**67 (26)**	**68 (26)**	**68 (26)**	**66 (25)**	**62 (24)**	**68 (20)**	**67 (18)**	**68 (17)**	**68 (18)**	**65 (19)**	**60 (18)**
**Social Functioning**	**82 (25)**	**80 (26)**	**83 (24)**	**84 (26)**	**82 (26)**	**79 (24)**	**83 (19)**	**80 (18)**	**83 (19)**	**84 (18)**	**81 (20)**	**76 (18)**
**Mental Health**	**75 (22)**	**76 (22)**	**77 (22)**	**78 (21)**	**78 (20)**	**75 (19)**	**74 (17)**	**75 (16)**	**76 (16)**	**79 (14)**	**78 (15)**	**75 (16)**

## References

[1] Ferlay J , Soerjomataram I , Dikshit R , et al. Cancer incidence and mortality worldwide: sources, methods and major patterns in GLOBOCAN 2012. Int J Cancer. 2015;136(5):E359‐386. 25220842 10.1002/ijc.29210

[2] Allemani C , Matsuda T , Di Carlo V , et al. Global surveillance of trends in cancer survival 2000–14 (CONCORD‐3): analysis of individual records for 37 513 025 patients diagnosed with one of 18 cancers from 322 population‐based registries in 71 countries. The Lancet. 2018;391(10125):1023‐1075. 10.1016/S0140-6736(17)33326-3 PMC5879496 29395269

[3] Soerjomataram I , Lortet‐Tieulent J , Parkin DM , et al. Global burden of cancer in 2008: a systematic analysis of disability‐adjusted life‐years in 12 world regions. The Lancet. 2012;380(9856):1840‐1850. 10.1016/S0140-6736(12)60919-2 23079588

[4] Lardas M , Liew M , van den Bergh RC , et al. Quality of life outcomes after primary treatment for clinically localised prostate cancer: a systematic review. Eur Urol. 2017;72(6):869‐885. 28757301 10.1016/j.eururo.2017.06.035

[5] Prabhu V , Lee T , McClintock TR , Lepor H . Short‐, Intermediate‐, and long‐term quality of life outcomes following radical prostatectomy for clinically localized prostate cancer. Rev Urol. 2013;15(4):161‐177. 24659913 PMC3922321

[6] Johansson E , Steineck G , Holmberg L , et al. Long‐term quality‐of‐life outcomes after radical prostatectomy or watchful waiting: the Scandinavian Prostate Cancer Group‐4 randomised trial. Lancet Oncol. 2011;12(9):891‐899. 21821474 10.1016/S1470-2045(11)70162-0

[7] Buzzoni C , Auvinen A , Roobol MJ , et al. Metastatic prostate cancer incidence and prostate‐specific antigen testing: new insights from the european randomized study of screening for prostate cancer. Eur Urol. 2015;68(5):885‐890. 25791513 10.1016/j.eururo.2015.02.042 PMC4982869

[8] Hugosson J , Roobol MJ , Månsson M , et al. A 16‐yr Follow‐up of the European Randomized study of Screening for Prostate Cancer. Eur Urol. 2019;76(1):43‐51. 30824296 10.1016/j.eururo.2019.02.009 PMC7513694

[9] Draisma G , Etzioni R , Tsodikov A , et al. Lead time and overdiagnosis in prostate‐specific antigen screening: importance of methods and context. J Natl Cancer Inst. 2009;101(6):374‐383. 19276453 10.1093/jnci/djp001 PMC2720697

[10] Fenton JJ , Weyrich MS , Durbin S , Liu Y , Bang H , Melnikow J . Prostate‐specific antigen‐based screening for prostate cancer: evidence report and systematic review for the US preventive services task force. JAMA. 2018;319(18):1914. 29801018 10.1001/jama.2018.3712

[11] Ilic D , Neuberger M , Djulbegovic M , Dahm P . Screening for prostate cancer. Cochrane Database Syst Rev. 2013;(1):CD004720. 23440794 10.1002/14651858.CD004720.pub3 PMC8406915

[12] Booth N , Rissanen P , Tammela TLJ , Määttänen L , Taari K , Auvinen A . Health‐Related Quality of Life in the Finnish Trial of Screening for Prostate Cancer. Eur Urol. 2014;65(1):39‐47. 23265387 10.1016/j.eururo.2012.11.041

[13] Taylor KL , Luta G , Miller AB , et al. Long‐term disease‐specific functioning among prostate cancer survivors and noncancer controls in the prostate, lung, colorectal, and ovarian cancer screening trial. J Clin Oncol. 2012;30(22):2768‐2775. 22734029 10.1200/JCO.2011.41.2767 PMC4166119

[14] Finne P , Stenman U‐H , Maattanen L , et al. The Finnish trial of prostate cancer screening: where are we now? BJU Int. 2003;92(Suppl 2):22‐26. 14983949 10.1111/j.1465-5101.2003.04397.x

[15] Hamoen EHJ , De Rooij M , Witjes JA , Barentsz JO , Rovers MM . Measuring health‐related quality of life in men with prostate cancer: A systematic review of the most used questionnaires and their validity. Urol Oncol. 2015;33(2):69.e19‐28. 10.1016/j.urolonc.2013.10.005 24433753

[16] Ware JE , Kosinski M , Dewey JE . How to Score Version 2 of the SF‐36 Health Survey. Lincoln, RI: QualityMetric Incorporated; 2000.

[17] Penson DF , Litwin MS , Lubeck DP , Flanders S , Pasta DJ , Carroll PR . Transitions in health‐related quality of life during the first nine months after diagnosis with prostate cancer. Prostate Cancer Prostatic Dis. 1998;1(3):134‐143. 12496906 10.1038/sj.pcan.4500228

[18] Moinpour CM , Darke AK , Donaldson GW , et al. Health‐related quality‐of‐life findings for the prostate cancer prevention trial. J Natl Cancer Inst. 2012;104(18):1373‐1385. 22972968 10.1093/jnci/djs359 PMC3529595

[19] Quan H , Li B , Couris CM , et al. Updating and validating the charlson comorbidity index and score for risk adjustment in hospital discharge abstracts using data from 6 countries. Am J Epidemiol. 2011;173(6):676‐682. 21330339 10.1093/aje/kwq433

[20] Mottet N , Bellmunt J , Bolla M , et al. Guidelines on prostate cancer. part 1: screening, diagnosis, and local treatment with curative intent. Eur Urol. 2017;71(4):618‐629. 27568654 10.1016/j.eururo.2016.08.003

[21] Graubard BI , Korn EL . Predictive margins with survey data. Biometrics. 1999;55(2):652‐659. 11318229 10.1111/j.0006-341x.1999.00652.x

[22] StataCorp . Stata Statistical Software: Release 14. College Station, TX: StataCorp LP; 2015.

[23] Kilpeläinen TP , Pogodin‐Hannolainen D , Kemppainen K , et al. Estimate of opportunistic prostate specific antigen testing in the finnish randomized study of screening for prostate cancer. J Urol. 2017;198(1):50‐57. 28104375 10.1016/j.juro.2017.01.048

[24] Auvinen A , Moss SM , Tammela TLJ , et al. Absolute effect of prostate cancer screening: balance of benefits and harms by center within the european randomized study of prostate cancer screening. Clin Cancer Res. 2016;22(1):243‐249. 26289069 10.1158/1078-0432.CCR-15-0941 PMC4951205

[25] Nevalainen J , Stenman U‐H , Tammela TL , et al. What explains the differences between centres in the European screening trial? A simulation study. Cancer Epidemiol. 2017;46:14‐19. 27889661 10.1016/j.canep.2016.11.005 PMC5415347

[26] Heijnsdijk EAM , Wever EM , Auvinen A , et al. Quality‐of‐life effects of prostate‐specific antigen screening. N Engl J Med. 2012;367(7):595‐605. 22894572 10.1056/NEJMoa1201637 PMC4982868

[27] Wolters T , Roobol MJ , Steyerberg EW , et al. The effect of study arm on prostate cancer treatment in the large screening trial ERSPC. Int J Cancer. 2010;126(10):2387‐2393. 19739124 10.1002/ijc.24870

